# Natural Pyrethrin-Induced Oxidative Damage in Human Liver Cells through Nrf-2 Signaling Pathway

**DOI:** 10.3390/toxics12040258

**Published:** 2024-03-30

**Authors:** Yun Yang, Xiaoyi Wei, Mengchao Ying, Haiyan Huang, Yijie Sha, Xinyu Hong, Ping Xiao, Gonghua Tao

**Affiliations:** 1 Shanghai Municipal Center for Disease Control & Prevention, Shanghai 200336, China; yangyun@scdc.sh.cn (Y.Y.); yingmengchao@scdc.sh.cn (M.Y.); shayijie@scdc.sh.cn (Y.S.); hongxinyu@scdc.sh.cn (X.H.); 2 State Environmental Protection Key Laboratory of Environmental Health Impact Assessment of Emerging Contaminants, Shanghai 200233, China; 3 Department of Food Science, College of Hospitality of Management, Shanghai Business School, Shanghai 200235, China; xiaoyi_wei@163.com; 4 Shenzhen Key Laboratory of Modern Toxicology, Shenzhen Center for Disease Control and Prevention, Shenzhen 518055, China; hhy424@126.com

**Keywords:** natural pyrethrins, oxidative stress, hepatotoxicity, Keap1/Nrf-2

## Abstract

Natural pyrethrins (NPs), one kind of bio-pesticide, have been widely used in organic agriculture and ecological environment studies. Studies have shown that NPs may affect the metabolism of rat liver and human hepatocytes; nevertheless, the toxic effects of NPs on the liver and the related mechanisms are still incompletely understood. In this research, we utilized three types of human liver cells to investigate the mechanism of NPs’ induction of oxidative stress. The results showed that NPs exhibit noteworthy cytotoxic effects on human liver cells. These effects are characterized by the induction of LDH release, mitochondrial collapse, and an increased production of ROS and MDA content, subsequently activating the Kelch-like ECH-associated protein 1/Nuclear factor erythroid 2- related factor 2 (Keap1/Nrf-2) pathway. The ROS inhibitor N-acetyl-L-cysteine (NAC) can alleviate ROS/Nrf2-mediated oxidative stress. In addition, the siRNA knockdown of Nrf-2 exacerbated the injury, including ROS production, and inhibited cell viability. In summary, the ROS-mediated Keap1/Nrf-2 pathway could be an important regulator of NP-induced damage in human liver cells, which further illustrates the hepatotoxicity of NPs and thereby contributes to the scientific basis for further exploration.

## 1. Introduction

Pesticides assume a crucial function in the regulation of agricultural pests, ensuring the quality and productivity of crops [1]. However, the extensive and improper utilization of pesticides results in an escalating array of environmental and public health repercussions, including but not limited to infertility, endocrine disruption, immunotoxicity, and even depressive symptoms [2]. This has garnered an increasing indication of the use of pesticides and raised concerns regarding human health. Natural pyrethrins (NPs) are extracted from the flowers of *cinerariaefolium*, encompassing six active ingredients, namely type I pyrethrins and type II pyrethrins [3]. The primary toxic mechanism of NPs is that they alter nerve functions by prolonging the opening of voltage-gated sodium channels, resulting in the production of neurotoxic effects that ultimately induce general exhaustion and eventual mortality in insects [4]. Although NPs are toxic for honey-bees, other pollinating insects, and fish, they are widely acknowledged for their minimal toxicity to mammals and limited environmental persistence, thus establishing them as the safest option. Due to their propensity for degradation being a major drawback, chemically synthesized analogues known as pyrethroids have been extensively investigated in order to enhance their efficacy, potency, and stability. Nevertheless, due to the widespread use of pyrethroids, which are recognized as posing risks to both the environment and human well-being, it is imperative to promote a heightened consciousness regarding the importance of environmental preservation and health safety [5].

In the contemporary era, the advancement of organic agriculture, sustainable pest management, and human safety considerations has prompted a shift in policies towards minimizing the utilization of synthetic pesticides [6]. Consequently, there has been a renewed focus among scientists on bio-pesticides, particularly NPs, which are permitted in green food and organic production as outlined in the NYT393-2013 guidelines and regulation (EC) No. 889/2008 (EC, 2008). This resurgence in interest has resulted in NPs capturing a significant 80% share of the botanical pesticides market [7]. Despite being regarded as environmentally safe and preferable to synthetic pesticides, bio-pesticides still present significant health hazards to non-target organisms, as evidenced by numerous studies [8]. While previous research primarily examined the toxicity of NPs in relation to human exposure, such as ocular, respiratory, and dermal symptoms [9], some studies also revealed that NPs are mitogenic CYP2B form inducers in rat liver [10], and recent studies have revealed ecotoxicologically relevant data demonstrating the toxicity of NPs to invertebrates and their ability to induce liver size changes and degeneration in zebrafish [11]. Furthermore, studies examining the cytotoxicity of NPs have demonstrated a reduction in total ATP levels and the induction of autophagy in neuroblastoma cell lines, as well as the initiation of programmed cell death in human liver cell lines and increased CYP2B and CYP3A forms in rat and human hepatocytes [12,13,14]. These findings collectively suggest that NPs pose a potential risk to both human health and the environment. 

Biological systems experience a continuous exposure to oxidative stressors, whether originating from internal processes or external sources. This exposure leads to the generation of elevated levels of ROSs as byproducts of metabolism, causing oxidative stress and disturbing the redox balance. Consequently, this disruption results in non-specific damage to DNA and proteins, ultimately leading to the development of various health disorders [15]. The Kelch-like ECH-associated protein 1/Nuclear factor erythroid 2-related factor 2 (Keap1/Nrf-2) pathway, known as a prominent cytoprotective response regulator, orchestrates the regulation of the antioxidant system by inducing a cascade of intracellular endogenous antioxidant enzymes, including glutathione peroxidase (GSH-Px), superoxide dismutase (SOD) antioxidant enzymes, NAD(P)H quinone oxidoreductase 1 (NQO1), and heme oxygenase-1 (HO-1) detoxification enzymes [16]. Nevertheless, the precise functions of Nrf-2 in the induction of oxidative stress by NPs remains uncertain. Hepatocyte cell lines, being more prone to injury, are commonly employed as in vitro models for assessing the detrimental impacts of xenobiotic metabolism, such as pesticide residues, on the liver [17]. In light of the lack of cytotoxicology data, we utilized the human liver cell line to investigate the effects of NP exposure and unravel the underlying mechanism caused by this widely used botanical pesticide.

## 2. Materials and Methods

### 2.1. Chemicals and Reagents

NPs (CAS: 8003-34-7, Sigma: 33739, consists of the following six active ingredients: 46.9% pyrethrinI, 23.0% pyrethrin II, 4.2% jasmolin I, 4.1% jasmolin II, 14.0% cinerin I and 7.8% cinerin II), MTT (3-(4,5-dimethylthiazol-2-yl)-2, 5-diphenyl-tetrazolium bromide), N-acetylcysteine (NAC), ROS, and Cytotoxicity Detection KitPLUS (LDH) were obtained from Sigma-Aldrich (St. Louis, MO, USA); ATP, SOD, and GSH-PX assay kits were obtained from BioAssay Systems (Hayward, CA, USA); MDA and JC-1 assay kits were purchased from Beyotime Institute of Biotechnology (Shanghai, China); TaKaRa minibest universal RNA extraction kit was obtained from Takara (Dalian, China); PowerUp™ SYBR™ Green was obtained from Thermo Scientific (Rockford, IL, USA); a stock solution of NPs (100 mg/mL) was prepared in DMSO and diluted in culture media to the desired concentrations.

### 2.2. Cell Culture and Treatment

The normal liver cell lines QSG7701 and WRL68 were purchased separately from Shanghai Yiyan Biological Technology Co., Ltd. (Shanghai, China) and Shanghai Chuan Qiu Biotechnology Co., Ltd. (Shanghai, China); another normal liver cell line, LO2, was provided by Fudan University, and the cells were all cultured in the recommended media, supplemented with 10% fetal bovine serum (FBS), 100 units/mL penicillin, and 100 μg/mL streptomycin, and maintained at 37 °C with 5% CO_2_.

For cell viability assay, three kinds of cells were treated with 0, 5, 10, 15, 20, 30, 40, 60, and 80 μg/mL of NPs for 24 and 48 h. For LDH measurements, three kinds of cells were treated with 0, 5, 10, 20, and 40 μg/mL of NPs for 24 h. For colony formation assay, three kinds of cells were treated with 0, 5, 10, 20, and 40 μg/mL of NPs for 6 and 24 h. For ATP assay, ROS and antioxidant enzymes level, MMP and QPCR analysis, three kinds of cells were treated with 0, 5, 10, 20, and 40 μg/mL of NPs for 6 h according to cell viability and proliferation assay. For NAC pretreatment and RNA interference assay, QSG7701 cells were treated with 0 and 40 μg/mL of NPs for 6 h.

### 2.3. Cell Viability Assay

Cell viability was evaluated by MTT assay. Briefly, different kinds of cells were seeded in 96-well plates until they reached a confluence of about 70–80%. Then, the medium was removed and replaced by fresh medium containing NPs which was dissolved in DMSO at various concentrations or 0.1% DMSO control. Following 24 or 48 h incubation, 20 μL of MTT solution (5 mg/mL) was added to each well incubated with cells for 4 h. Subsequently, the medium with MTT was removed and 150 μL DMSO was added to dissolve the formazan crystals, then the medium shaken for 5 min. Finally, the absorbance was measured at 570 nm using a microplate reader (SPECTROstar Nano, BMG, GER). The inhibitory percentage of NPs was calculated, and the IC50 value was determined. 

### 2.4. LDH Measurements

Different kinds of cells were seeded in 96-well plates until they reached a confluence of about 70–80%. Then, the medium was removed and replaced by fresh medium containing NPs. After 24 h incubation, 100 µL of reaction mixture was added to the supernatants and incubated 8 min at from 15 to 25 °C. Then, 50 µL of stop solution was added and the absorbance was recorded at 490 nm using a micro plate spectrophotometer system; results are presented as percentage of control values.

### 2.5. Colony Formation Assay

Different kinds of cells were seeded in 9.6 cm^2^ plates for about 10 h. Then, the medium was removed and replaced by fresh medium containing NPs. After 6 and 24 h incubation, cells were incubated for 10–14 days, then fixed and stained with Giemsa staining solution. Only colonies containing more than 50 cells were counted.

### 2.6. ATP Assay

The EnzyLightTM ATP assay kit was used for ATP assay following the manufacturer’s instruction, and the luminescence was proportional to the amount of ATP present. Briefly, different kinds of cells were seeded in 96-well plates until they reached a confluence of about 70–80%. Then, the medium was removed and replaced by fresh medium containing NPs. After 6 h incubation, 90 μL of reconstituted reagent was added to each well, and the luminescence was read in a microplate plate reader within 1 min. 

### 2.7. Measurement of ROS Level

The ROS level was analyzed with ROS kit according to the optimized manufacturer’s instructions. Briefly, different kinds of cells were seeded in 96-well black plate until they reached a confluence of about 70–80%. Then, the medium was removed and replaced by fresh medium containing NPs. After 6 h incubation, 100 mL/well of master reaction mix was added into the cell plate and incubated for from 30 min to one hour. Then, the ROS level was detected by measuring the fluorescence with fluorescent microplate reader (spark, Tecan, Männedorf, Switzerland) at an excitation wavelength of 640 nm and at an emission wavelength of 675 nm.

### 2.8. Measurement of Antioxidant Level

The oxidative stress parameters, including activities of antioxidant, associated enzymes, and lipid peroxides, were measured. Commercial kits were used for the measurements of activities of antioxidant enzyme superoxide dismutase (SOD), glutathione peroxidase (GSH-Px), and the lipid peroxidation malondialdehyde (MDA) following the manufacturer’s instructions. In brief, different kinds of cells were seeded in 6-well plates until they reached a confluence of about 70–80%. Then, the medium was removed and replaced by fresh medium containing NPs. After 6 h incubation, the cells were resuspended and centrifuged. The activity of antioxidant level in the supernatant was measured, and the value was normalized to the protein level.

### 2.9. Measurement of Mitochondrial Membrane Potential (MMP)

The effects of NPs on MMP were detected using mitochondrial-specific cationic dye JC-1. In brief, different kinds of cells were seeded in 24-well plates until they reached a confluence of about 70–80%. Then, the medium was removed and replaced by fresh medium containing NPs. After 6 h incubation, the cells were preloaded with JC-1 for 30 min, and then rinsed with freshly prepared phosphate-buffered saline (PBS, pH 7.4). Fluorescence of JC-1 monomers (excitation wavelength of 480 ± 20 nm, emission wavelength of 527 ± 15 nm) and aggregates (excitation wavelength of 538 ± 22.5 nm, emission wavelength of >590 nm) was photographed and analyzed for red/green fluorescence by fluorescence microscopy (Leica, DM3000).

### 2.10. Quantitative Real-Time PCR Analysis

The extraction of total RNA from the cells was extracted using RNA extraction kit in accordance with the instructions provided by the manufacturer. In brief, different kinds of cells were seeded in 6-well plates until they reached a confluence of about 70–80%. Then, the medium was removed and replaced by fresh medium containing NPs. After 6 h incubation, cells were exposed to RNA extraction kit to extract total RNA and the RNA concentration was determined by NanoDrop 2000 (Thermo Scientific, USA), and complementary DNA (cDNA) was reverse-transcribed by RNA utilizing reverse transcription kit using PCR (GeneAmp PCR system 9700, Thermo Scientific, USA). Then, the cDNA templates were quantified with PowerUp™ SYBR™ Green using real-time PCR (QuantStudio 7 Flex, Thermo Scientific, USA). The results were calculated using 2^−ΔΔCt^. The PCR primers were as follows: Nrf-2 ACGGTATGCAACAGGACATTGAGC (forward) and TTGGCTTCTGGACTTGGAACCATG (reverse); Keap1 ATTCAGCTGAGTGTTACTACCC (forward) and CAGCATAGATACAGTTGTGCAG; HO-1 CCTCCCTGTACCACATCTATGT (forward) and GCTCTTCTGGGAAGTAGACAG (reverse); NQO1 AGTATCCTGCCGAGTCTGTTCTGG (forward) and AATATCACAAGGTCTGCGGCTTCC (reverse); GAPDH CAGGAGGCATTGCTGATGAT (forward) and GAAGGCTGGGGCTCATTT (reverse). 

### 2.11. NAC Pretreatment

After incubation with NAC (1 mM) for 2 h, the cells were cultured with NPs for another 6 h. Then, ROS and related genes were measured by the above-described methods.

### 2.12. RNA Interference Assay

Negative control (NC) and Nrf-2 small interfering RNA (siRNA) were synthesized by GenePharma Co., Ltd. (Shanghai, China), and performed using Lipofectamine RNAiMAX reagent (Invitrogen, California, USA) in cells. The sequences of siRNA were as follows: NC 5′-UUCUCCGAACGUGUCACGUTT ACGUGACACGUUCGGAGAATT-3′; Nrf-2 5′-GCCCAUUGAUGUUUCUGAUTT AUCAGAAACAUCAAUGGGCTT-3′.

### 2.13. Statistical Analysis

Data were expressed as the form of mean ± standard deviation (SD). Results of three individual experiments were used statistically by one-way ANOVA followed by Dunnett’s test to determine the differences between groups. A *p*-value < 0.05 was considered a statistically significant difference. 

## 3. Results

### 3.1. Subsection

#### 3.1.1. NP Inhibited Viability in Liver Cells

**Figure 1:**
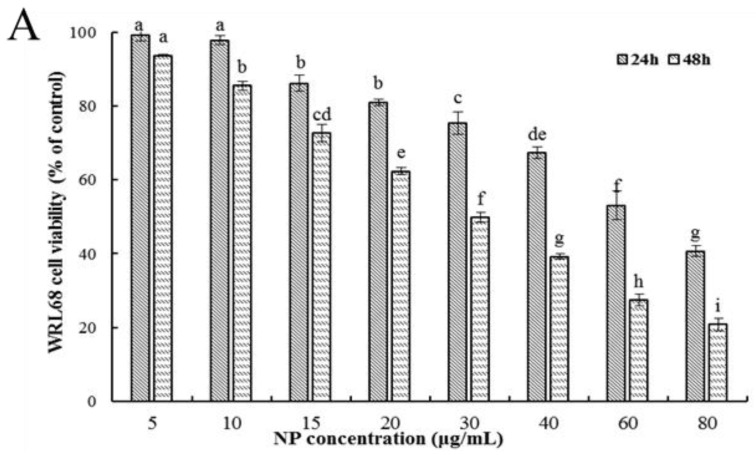

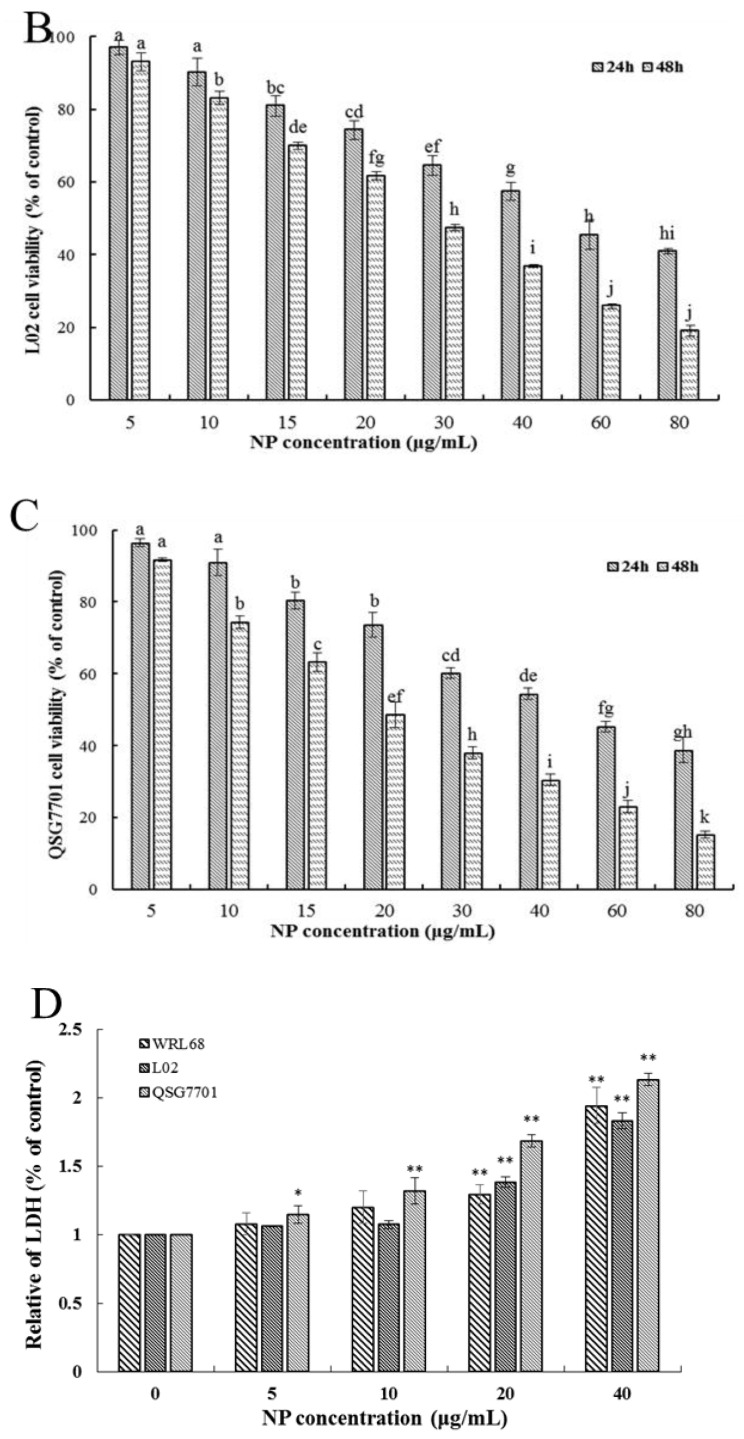
Effects of NP on the activity of hepatocytes. Cell activities (**A**–**C**) and LDH activity (**D**) of NPs on WRL68, L02, and QSG7701 cells. The letters indicated significant differences between any two groups (* *p* ≤ 0. 05, ** *p* ≤ 0. 01).

Firstly, the ability of NPs to inhibit cellular viability was assessed by an MTT assay using three types of human liver cells. As shown in Figure 1A–C, after being exposed to a series of concentrations of NPs for 24 and 48 h, concentration- and time-dependent reductions in the viability of the three types of human liver cells were observed, which indicated a significant cytotoxicity of NPs. The respective IC50 values of the WRL68, L02, and QSG7701 cells for 24 h were approximately 51.42, 42.82, and 41.15 μg/mL, and for 48 h they were approximately 28.62, 27.14, and 21.5 μg/mL, which indicated that the QSG7701 and L02 cell lines appear to be more sensitive than the WRL68 line. To study the cell membrane-damaging potential of NPs against three types of human liver cells, the LDH leakage into the supernatant of culture following 24 h treatment was measured (Figure 1D). The result showed that treatment with different concentrations of NPs produced a significant increase in the LDH levels in comparison to the control group.

#### 3.1.2. NP Decreased Colony Formation in Liver Cells

**Figure 2:**
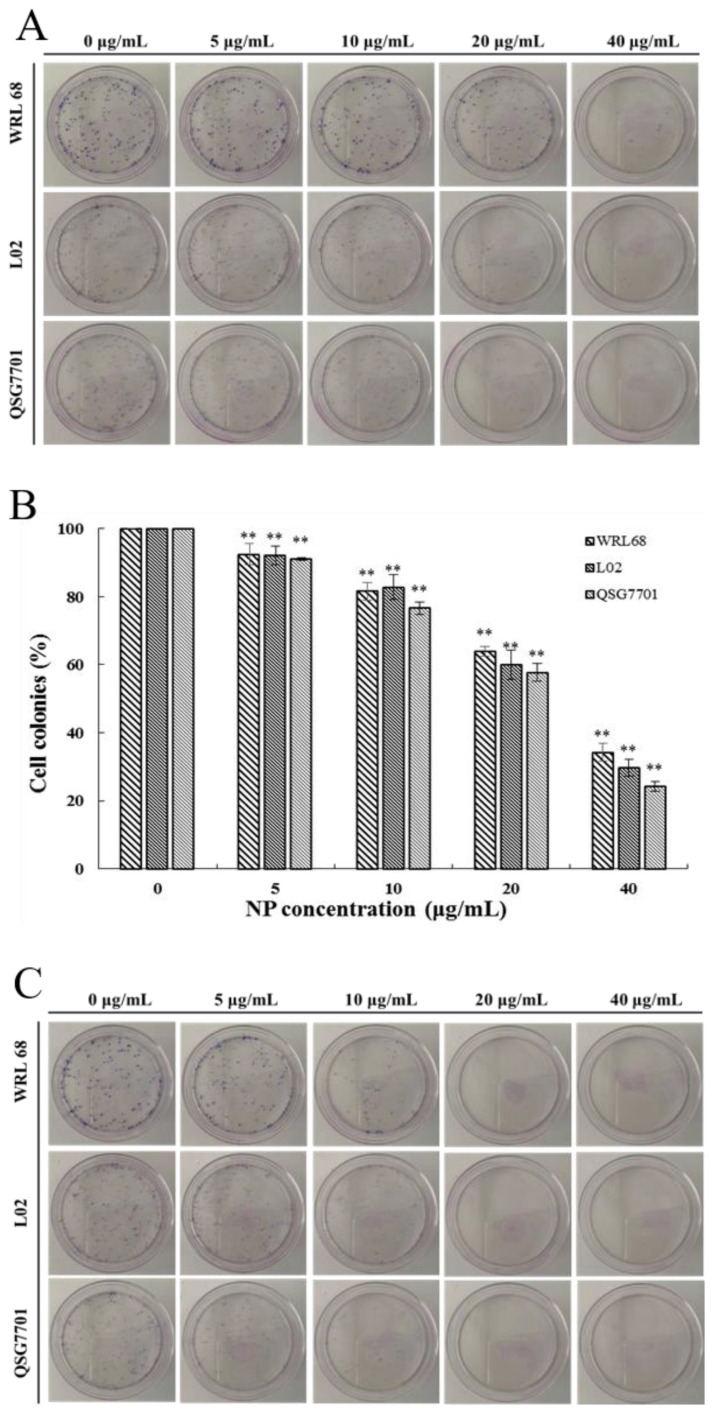

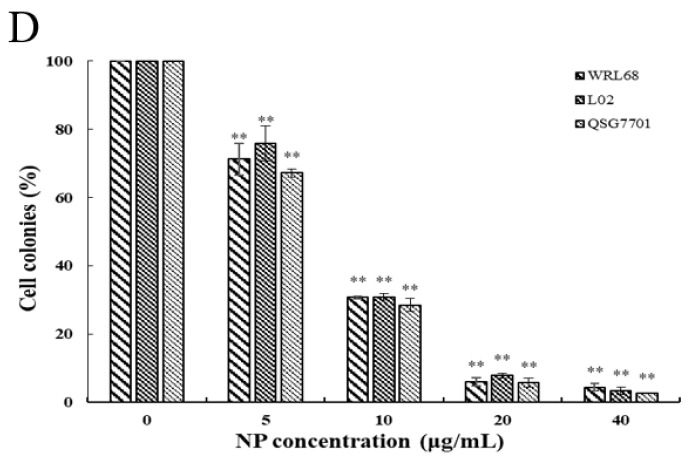
Inhibition of NPs on WRL68, L02, and QSG7701 cells for 6 and 24 h (**A**,**C**): colony formation photographs for 6 and 24 h; (**B**,**D**): percentage of colonies for 6 and 24 h (** *p* ≤ 0.01).

The relatively short-term MTT results were supplemented by long-term colony formation assays, which were adopted to detect whether the NPs would inhibit the formation of clonal spheres of liver cells. After being exposed to different concentrations of NPs for 6 and 24 h, the ability of the three types of liver cells to spawn a colony of descendants was determined during the following next two weeks, as shown in Figure 2A,C. The results showed that the NPs abolished the colony formation in a concentration-dependent manner compared to the control cells, and the growth-inhibitory effect of NPs appears to be more pronounced in QSG7701 than in other cells (Figure 2B,D). These observations indicated that NPs have antiproliferative effects on liver cells.

#### 3.1.3. NP Inhibited Mitochondrial Dysfunction in Liver Cells

**Figure 3:**
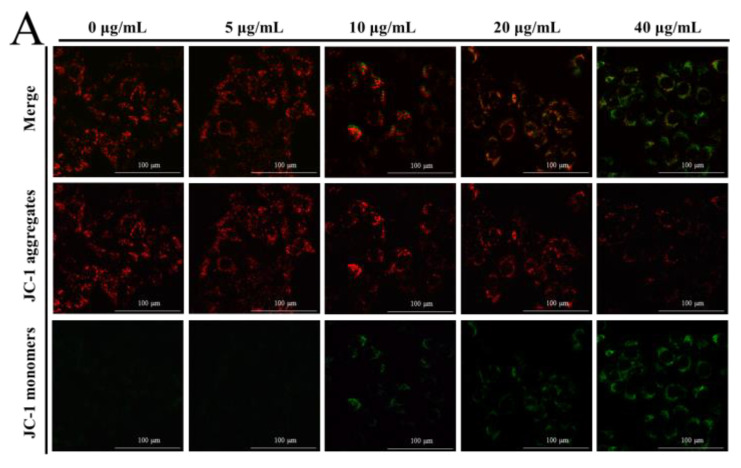

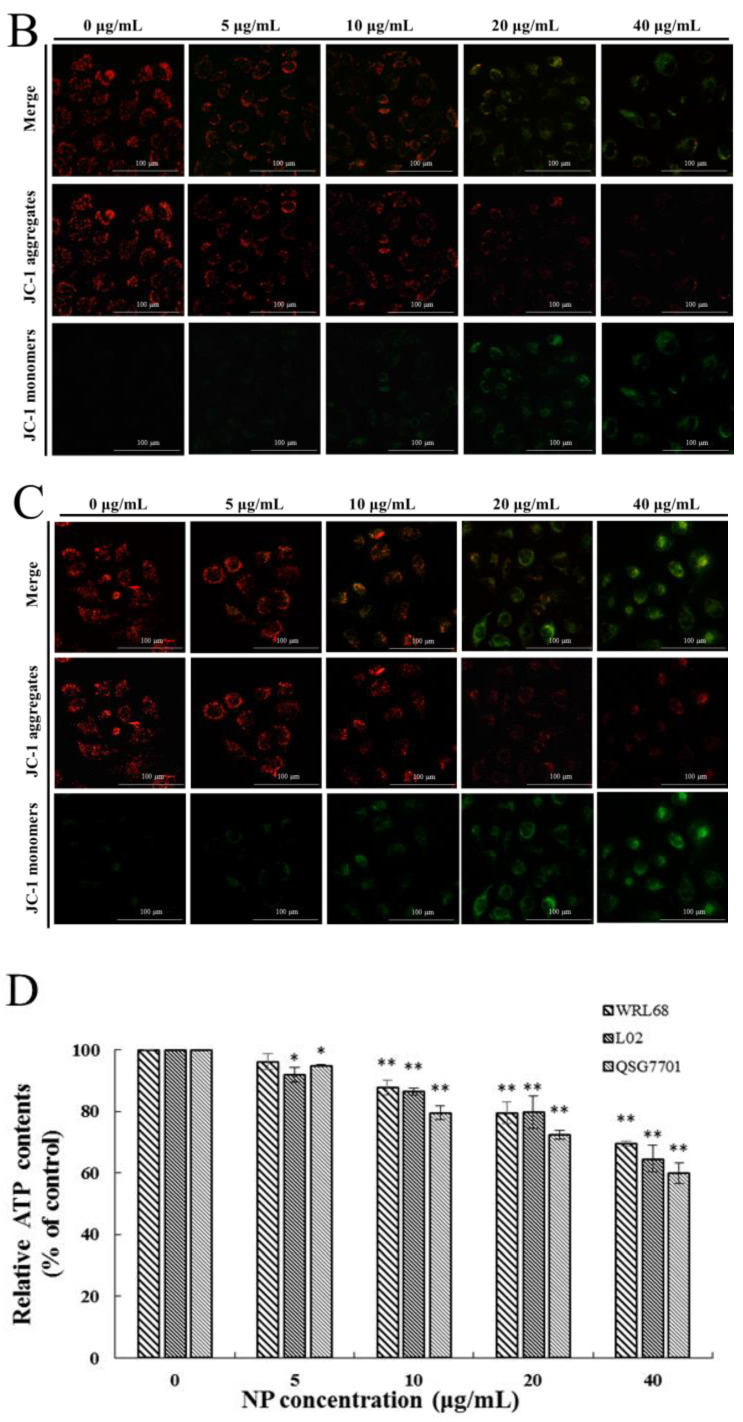
Effects of NPs on mitochondria of hepatocytes. MMP (**A**–**C**) and ATP activity (**D**) of NPs on WRL68, L02, and QSG7701 cells (* *p* ≤ 0.05, ** *p* ≤ 0.01).

The MMP was determined by JC-1 dye to elucidate the effect of NPs on the integrity of the mitochondrial membrane, through the red to green ratio parallel to the control cells. When the dye accumulates in the mitochondria of low membrane potential in monomer form, it emits a green fluorescence following excitation by blue fluorescence. At high membrane potentials, it forms ‘J-aggregates’, which emit a red fluorescence following excitation by green light. In our results, a concentration-dependent reduction in red fluorescence and increase in the green fluorescence were observed with NP treatment, as shown in Figure 3A–C, which undoubtedly revealed the loss of the MMP. This indicated a remarkable cytotoxic activity, which was consistent with the actions in the MTT and colony formation assays. The ATP level is a direct indicator of energy status, which was observed through ATP detection kits. In this ATP-luminescent reaction, we detected the intracellular levels of ATP in liver cells, which were noticeably reduced after culturing with NPs for 6 h compared to the control group (Figure 3D).

#### 3.1.4. NP Induced Oxidative Stress Production in Liver Cells

**Figure 4:**
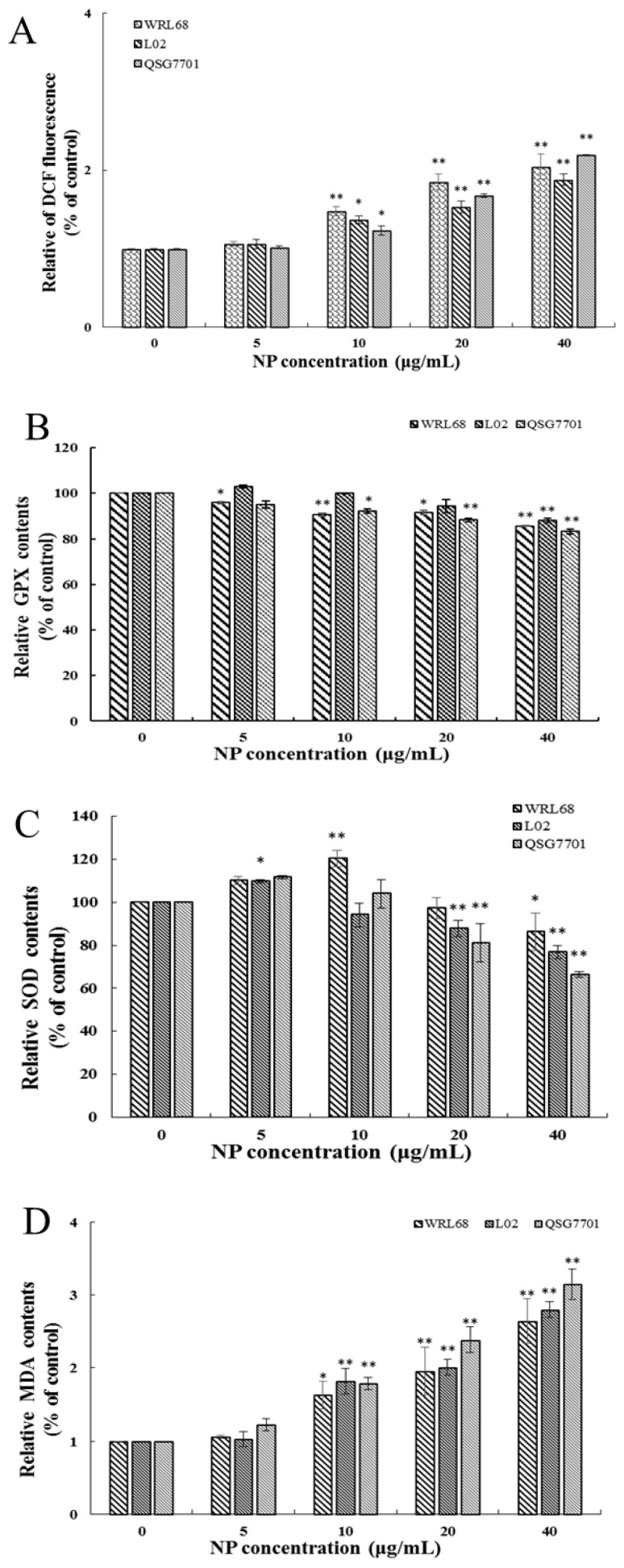
Effects of NPs on oxidative damage of hepatocytes. Effects of ROS (**A**), GPX (**B**), SOD (**C**), and MDA (**D**) activity of WRL68, L02, and QSG7701 cells (* *p* ≤ 0.05, ** *p* ≤ 0.01).

ROS generation was investigated using the fluorescent dye DCFH-DA. The fluorescence intensities of DCF signals in the cells showed significant elevation after NP treatment in a concentration-dependent fashion (Figure 4A). The productions of SOD, GSH-Px activities, and MDA levels were measured to investigate the effect of NPs on cellular oxidative stress in liver cells. After NP administration, the expression change in GSH-Px was decreased, and the antioxidative enzymes and SOD concentrations exhibited a trend of first increasing and then decreasing significantly in a concentration-dependent manner (Figure 4B,C). Figure 4D shows that the amount of MDA was remarkably increased in NP-exposed groups.

#### 3.1.5. NP Activated the Nrf-2 Pathway in Liver Cells

**Figure 5:**
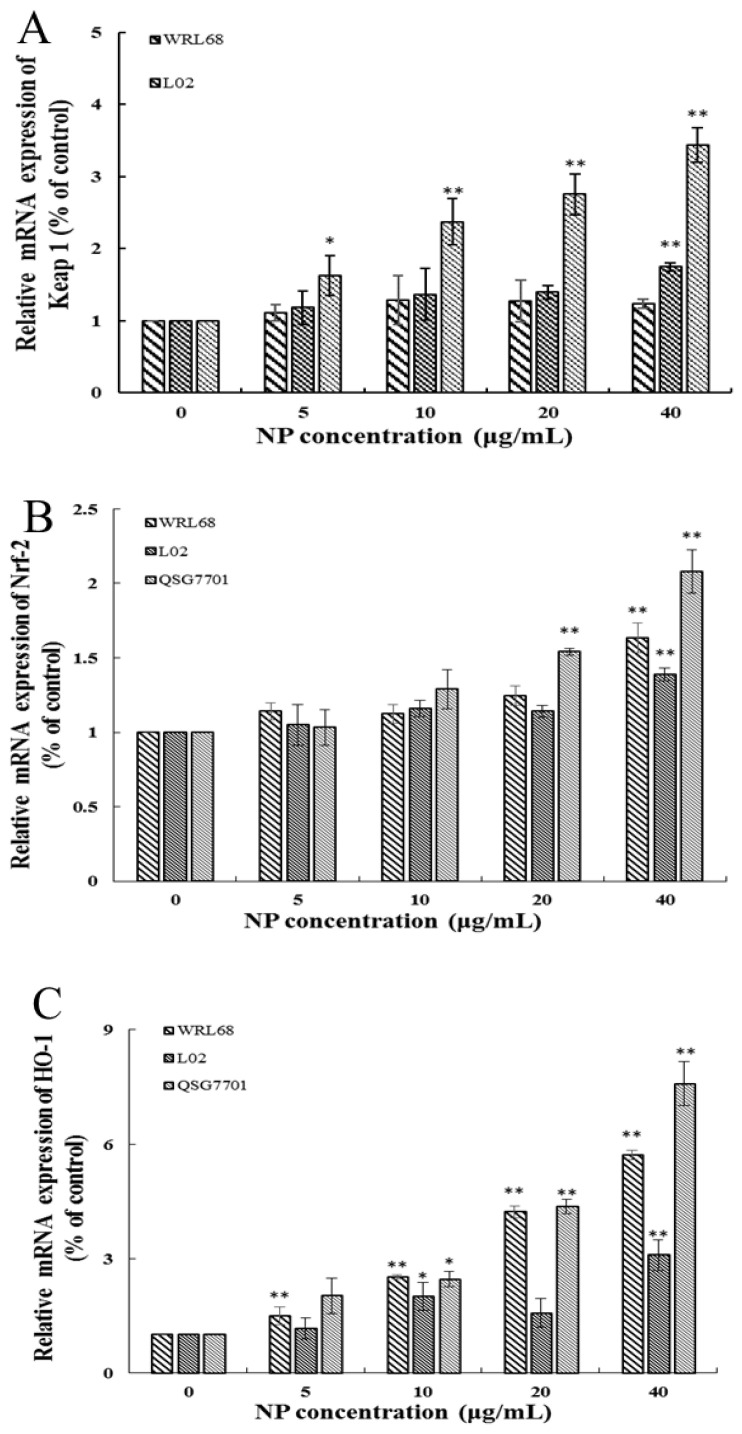

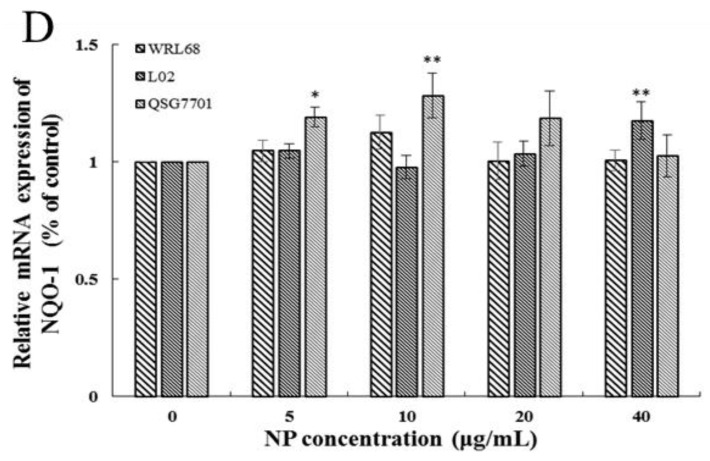
Effects of NPs on Nrf-2 signaling pathway. Detection of Keap1 (**A**), Nrf-2 (**B**), HO-1 (**C**), and NQO-1 (**D**) mRNA levels in WRL68, L02, and QSG7701 cells (* *p* ≤ 0.05, ** *p* ≤ 0.01).

The Keap1/Nrf-2 signaling pathway is widely recognized as the “power switch” for oxidative stress. We found that the mRNA levels of Keap1 and Nrf-2 levels in the three types of human liver cells were upregulated in a concentration-dependent manner in response to NP treatment (Figure 5A,B). We also detected the expression of Nrf-2 downstream phase II antioxidant enzymes, such as HO-1 and NQO1, which reduce oxidative stress injury. And the results showed that the downstream enzyme HO-1 was significantly elevated in the NP-exposed groups; however, the expression change in the NQO-1 mRNA level exhibited a slight increase but no significant alteration (Figure 5C,D).

#### 3.1.6. NAC Alleviate NP Induced Nrf-2 Pathway by Reducing ROS in Liver Cells

**Figure 6:**
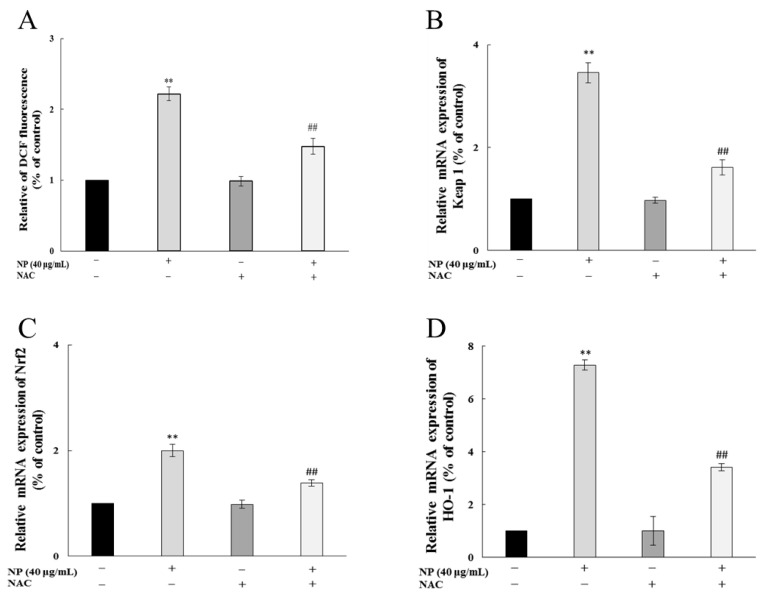
NAC alleviated NP-induced Nrf-2 pathway by reducing ROSs in QSG7701 cells. Effects of ROSs (**A**), Keap1 (**B**), Nrf-2 (**C**), and HO-1 (**D**) mRNA levels in QSG7701 cells exposed to 40 μg/mL NP with and without NAC treatment (** *p* ≤ 0.01 vs. control. ## *p* < 0.01 vs. the same dose of NPs group).

After ROS scavenger NAC treatment, the ROS production in QSG7701 cells caused by the NPs was significantly inhibited (Figure 6A), and the rise in the expression of the antioxidant genes Keap1, Nrf-2, and HO-1 was mitigated (Figure 6B–D), which indicated that NAC effectively inhibited the NP-induced Nrf-2 pathway by reducing the oxidative stress of the human liver cells.

#### 3.1.7. Nrf-2 Regulated NP-Induced ROS in Liver Cells

**Figure 7:**
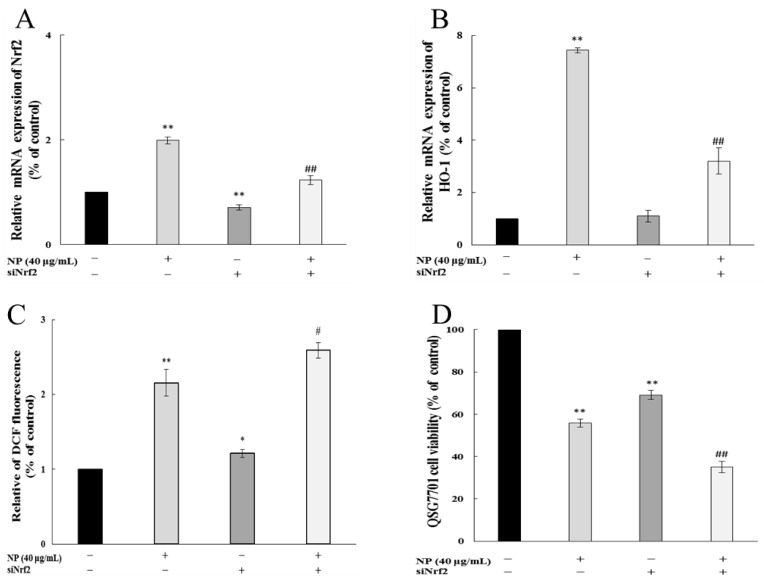
Nrf-2 regulates NP-induced ROSs and Nrf-2 pathway in QSG7701 cells. Effects of Nrf-2 (**A**) and HO-1 (**B**) mRNA levels, ROSs (**C**), and cell activities (**D**) in QSG7701cells exposed to 40 μg /mL NPs with and without Nrf-2 siRNA treatment (* *p* ≤ 0.05, ** *p* ≤ 0.01 vs. control. # *p* < 0.05, ## *p* < 0.01 vs. the same dose of NP group).

To detect whether Nrf-2 played a role in the oxidative stress in cells, we examined the impact of NPs on ROSs and cell viability by Nrf-2 siRNA. Figure 7A shows that the siRNA knockdown of the Nrf-2 model was successfully established. And the inhibition of Nrf-2 further increased the NP-induced ROS production and decreased the mRNA levels of HO-1 (Figure 7B,C). The MTT results showed that the NPs significantly decreased the cell viability through Nrf-2 siRNA (Figure 7D).

## 4. Discussion

Xenobiotics, such as pesticides, have the potential to accumulate in organs, leading to bioaccumulation. This accumulation can trigger the generation of ROSs, resulting in the production of oxidative stress and the induction of DNA breaks. Ultimately, these processes can lead to toxicity and the development of carcinogenic effects [18]. Due to the adverse effects of synthetic pesticides on both the environment and human health, the utilization of bio-pesticide agents has become imperative. These agents are considered safer alternatives as they are derived from naturally occurring substances [19]. The exposure to NPs, considered a promising bio-pesticide, has garnered significant attention due to its recognized safety and potential for reducing the risk of toxicity in humans [20]. The liver is widely recognized as a highly susceptible target tissue for toxicity caused by environmental chemicals, rendering it more vulnerable to injury [21]. Therefore, this study utilized three types of human liver cells through a series of experimental procedures to investigate the mechanism of NPs’ induction of oxidative stress. 

Firstly, the MTT method was employed to preliminarily evaluate the toxicity of NPs. The findings revealed a decline in the viability of three types of human liver cell in a concentration- and time-dependent manner upon exposure to NPs. Additionally, the cells exhibited signs of separation and irregular morphological changes. The IC50 values for the cells at 24 and 48 h were approximately 51.42, 42.82, and 41.15 μg/mL, and 28.62, 27.14, and 21.5 μg/mL, indicating that the QSG7701 and L02 cell lines displayed greater sensitivity compared to the WRL68 cell line. In previous studies, variations in IC50 values were observed among QSG7701, HepG2, and SHSY5Y cell lines, potentially attributable to species disparities and experimental conditions, including the environmental temperature, exposure duration, and cellular morphological characteristics that influence their cytotoxicity [12,13,22]. In addition to assessing mitochondrial dehydrogenase impairment, we also examined the loss of membrane integrity through the LDH leakage assay, which serves as an additional indicator of cellular damage, specifically the disruption of plasma membrane integrity [23]. Yi Han et al. posited that LDH may serve as a predictive risk factor for the severity of COVID-19 [24]. In our study, we observed a concentration-dependent increase in LDH release from three types of cells following treatment with NPs, suggesting that these compounds may compromise cell membrane integrity and alter cellular permeability. Furthermore, the detrimental impact of NPs on liver cells was corroborated by the colony formation assay. The continuous proliferation capacity of cells is essential for maintaining cellular integrity. As depicted in Figure 2, the concentration-dependent inhibition of colony formation in three liver cell lines by NPs suggests their cytotoxic effect, hindering cellular proliferation and colony formation. Consequently, the aforementioned data strongly indicate the significant cytotoxicity of NPs on human hepatocytes.

ATP, being the fundamental energy converter, is produced through oxidative phosphorylation in the mitochondria and glycolysis in the cytosol. When subjected to external environmental stress, the inhibition of mitochondrial oxidative phosphorylation in cells occurs, resulting in a reduction in ATP synthesis [25]. In our ATP-luminescent reaction, the treated groups exhibited a significant concentration-dependent decrease in ATP activity compared to the control group, suggesting that NPs have the potential to disrupt the regular energy metabolism of liver cells. The ATP level serves as both a direct indicator of the energy state and an additional indicator of mitochondrial function. Typically, a reduction in ATP signifies compromised mitochondrial function, which in turn impacts regular cellular physiological processes [26]. The maintenance of energy production, as well as the activity and normal physiological functions of mitochondria, relies on the MMP. Disruptions to the transmembrane potential can have significant repercussions on mitochondrial respiration and energy production. The findings of our study confirm that the exposure time to NPs exacerbates the decrease in MMP levels.

Moreover, mitochondria, which serve as crucial markers of cellular impairment, also represent a significant origin of intracellular ROS production. A plethora of evidence suggests that ROSs act as “redox messengers” in intracellular signaling and regulatory pathways, while the buildup of ROSs leads to oxidative harm, resulting in detrimental effects on organelle functionality and a strong association with metabolic disorders and apoptotic cell demise. Thus, in the present study, the levels of ROSs were assessed in three liver cell types. Our findings indicate that NPs induced a concentration-dependent elevation in ROS levels. This suggests that NPs exert a detrimental effect on the hepatic antioxidant defense system, ultimately leading to oxidative stress.

As a crucial cellular defense mechanism, the Keap1/Nrf-2 signaling pathway regulates oxidative stress and preserves redox equilibrium [27]. Upon encountering oxidative stress, Nrf-2, acting as a pivotal sensor, dissociates from Keap1 and translocates into the nucleus where it binds to the antioxidant response element (ARE) promoter. This interaction triggers the induction of phase II detoxification enzymes (e.g., HO-1 and NQO1) and antioxidant enzymes (e.g., GSH-Px and SOD), which collectively work to counteract oxidative stress-induced damage and effectively uphold intracellular redox homeostasis [28]. Additionally, MDA, a biomarker of lipid peroxidation, is closely associated with cellular harm resulting from oxidative stress [29]. Yang et al. have provided evidence of cellular damage induced by the pyrethroid deltamethrin through the mediation of the Nrf-2/HO-1 signaling pathway, which is linked to oxidative stress [30]. Diogo et al. assessed the acute and sub-chronic impacts of NPs on ecotoxicological effects. The researchers observed an elevation in the activities of SOD, CAT, GSH-Px, and lipid peroxidation, indicating the susceptibility of non-target organisms to NPs [31]. 

Our study demonstrated that NPs effectively activated the mRNA levels of Nrf-2, Keap 1, and HO-1, decreased the viability of the GSH-Px and SOD, and raised the content of MDA, which indicated that the NPs were activating the antioxidant system of human liver cells. Similarly, Guo et al. demonstrated microplastics- and nanoplastics-induced ROS could activate the Nrf-2 pathway in human liver cells [32]. Based on the above results, we wonder whether NP-induced liver injury may be due to the inability of the upregulated Nrf-2 to fully resist excess ROSs in liver cells. We chose the most sensitive liver cell line, QSG7701, to detect whether NP-caused Keap1/Nrf-2 pathway activated is dependent on intracellular ROSs. Firstly, NP exposure significantly promoted the Nrf-2 pathway, while the inhibition of ROSs by NAC significantly alleviates the NP-induced up-regulation of Keap1, Nrf-2, and HO-1 expression. Besides, a Nrf-2 siRNA was transfected into QSG7701 cells, and the knockdown of Nrf-2 markedly aggravated ROS production and HO-1 expression, and significantly decreased the cell viability under NP exposure. Therefore, we believe that the activation of the Keap1/Nrf-2 pathway mediated by ROSs is critical in NP-induced liver toxicity. Previous studies have shown that ROS elimination by NAC could inhibit the Nrf-2/Keap1 activation, and silencing Nrf-2 significantly inhibited autophagy induced by PM2.5 [33]. However, the mechanisms of autophagy caused by NPs are unclear and worthy of further exploration.

## 5. Conclusions

In conclusion, the current investigation has determined that NPs exhibit noteworthy cytotoxic effects on three types of human liver cells. These effects are characterized by the induction of mitochondrial collapse and the increased production of ROSs, which subsequently activate the Keap1/Nrf-2 signaling pathway. This pathway leads to the upregulation of Keap1, Nrf-2, and HO-1 expression, thereby mitigating oxidative damage. Our study suggested that the ROS-mediated Keap1/Nrf-2 pathway could be an important regulatory of NP-induced damage in human liver cells, which provided a new perspective on the mechanism of toxicity induced by NP exposure. Historically, emphasis has predominantly been placed on the health hazards associated with chemical pesticides, with the potential risks posed by biological pesticides often being overlooked. Consequently, further investigation into the toxicity of NPs will be a primary area of focus in our forthcoming research endeavors.

## Data Availability

The original contributions presented in the study are included in the article, further inquiries can be directed to the corresponding authors.

## References

[1] Alshemmari H., Al-Shareedah A.E., Rajagopalan S., Talebi L.A., Hajeyah M. (2021). Pesticides driven pollution in Kuwait: The first evidence of environmental exposure to pesticides in soils and human health risk assessment. Chemosphere.

[2] Cancino J., Soto K., Tapia J., Muñoz-Quezada M.T., Lucero B., Contreras C., Moreno J. (2023). Occupational exposure to pesticides and symptoms of depression in agricultural workers. A systematic review. Environ. Res..

[3] Zhang H., Xie Z.F., Peng Y.M., Xie A., Fu C.L., Zheng D.Y., Cai Z.W., Zhong J., Ming Q., Li M. (2023). PARP1 promotes NLRP3 activation via blocking TFEB-mediated autophagy in rotenone-induced neurodegeneration. Ecotoxicol. Environ. Saf..

[4] Markham T.E., Duggan P.J., Johnston M.R. (2006). Investigating the Diels-Alder reactivity of the natural pyrethrins. Tetrahedron.

[5] Zhu Q.Y., Yang Y., Zhong Y.Y., Lao Z.T., O’Neill P., Hong D., Zhang K., Zhao S. (2020). Synthesis, insecticidal activity, resistance, photodegradation and toxicity of pyrethroids (A review). Chemosphere.

[6] Ochieng L.O., Ogendo J.O., Bett P.K., Nyaanga J.G., Cheruiyot E.K., Mulwa R.M.S., Arnold S.E.J., Belmain S.R., Stevenson P.C. (2022). Field margins and botanical insecticides enhance Lablab purpureus yield by reducing aphid pests and supporting natural enemies. J. Appl. Entomol..

[7] Mossa A.H., Mohafrash S.M.M., Chandrasekaran N. (2018). Safety of Natural Insecticides: Toxic Effects on Experimental Animals. Biomed. Res. Int..

[8] Diao L., Tang N., Zhang C., Cheng J.G., Zhang Z.H., Wang S.Y., Wu C., Zhang L., Tao L., Li Z. (2021). Avermectin induced DNA damage to the apoptosis and autophagy in human lung epithelial A549 cells. Ecotoxicol. Environ. Saf..

[9] Singh S., Mukherjee A., Jaiswal D.K., Pereira A.P., Prasad R., Sharma M., Kuhad R.C., Shukla A.C., Verma J.P. (2022). Advances and future prospects of pyrethroids: Toxicity and microbial degradation. Sci. Total Environ..

[10] Price R.J., Walters D.G., Finch J.M., Gabriel K.L., Capen C.C., Osimitz T.G., Lake B.G. (2007). A mode of action for induction of liver tumors by Pyrethrins in the rat. Toxicol. Appl. Pharm..

[11] Lu J., Yang Y., Zhu L.H., Li M., Xu W.P., Zhang C., Cheng J.G., Tao L., Li Z., Zhang Y. (2022). Exposure to environmental concentrations of natural pyrethrins induces hepatotoxicity: Assessment in HepG2 cell lines and zebrafish models. Chemosphere.

[12] Yang Y., Zhang Y., Gao J.F., Xu W.P., Xu Z.P., Li Z.P., Cheng J.G., Tao L. (2020). Pyrethrum extract induces oxidative DNA damage and AMPK/mTOR-mediated autophagy in SH-SY5Y cells. Sci. Total Environ..

[13] Yang Y., Gao J.F., Zhang Y., Xu W.P., Hao Y.W., Xu Z.P., Tao L.M. (2018). Natural pyrethrins induce autophagy of HepG2 cells through the activation of AMPK/mTOR pathway. Environ. Pollut..

[14] Price R.J., Giddings A.M., Scott M.P., Walters D.G., Capen C.C., Osimitz T.G., Lake B.G. (2008). Effect of Pyrethrins on cytochrome P450 forms in cultured rat and human hepatocytes. Toxicology.

[15] Maity S., Guchhait R., De S., Pramanick K. (2023). High doses of nano-polystyrene aggravate the oxidative stress, DNA damage, and the cell death in onions. Environ. Pollut..

[16] Cheng C.J., Zhang J.L., Liu K.X., Xu Y.Y., Shen F.K., Han Y.Q., Hou Y.Y., Zhang T., Bai G. (2023). Ginsenoside CK targeting KEAP1-DGR/Kelch domain disrupts the binding between KEAP1 and NRF2-DLG motif to ameliorate oxidative stress damage. Phytomedicine.

[17] Derakhshesh N., Salamat N., Movahedinia A., Hashemitabar M., Bayati V. (2019). Exposure of liver cell culture from the orange-spotted grouper, Epinephelus coioides, to benzo[a]pyrene and light results in oxidative damage as measured by antioxidant enzymes. Chemosphere.

[18] Kalyabina V.P., Esimbekova E.N., Kopylova K.V., Kratasyuk V.A. (2021). Pesticides: Formulants, distribution pathways and effects on human health—A review. Toxicol. Rep..

[19] Khursheed A., ARather M., Jain V., Wani A.R., Rasool S., Nazir R., Malik N.A., Majid S.A. (2022). Plant based natural products as potential ecofriendly and safer biopesticides: A comprehensive overview of their advantages over conventional pesticides, limitations and regulatory aspects. Microb. Pathog..

[20] Lybrand D.B., Xu H.Y., Last R.L., Pichersky E. (2020). How Plants Synthesize Pyrethrins: Safe and Biodegradable Insecticides. Trends Plant Sci..

[21] Akinmoladun A.C., Oladejo C.O., Josiah S.S., Famusiwa C.D., Ojo O.B., Olaleye M.T. (2023). Catechin, quercetin and taxifolin improve redox and biochemical imbalances in rotenone-induced hepatocellular dysfunction: Relevance for therapy in pesticide-induced liver toxicity?. Pathophysiology.

[22] Yang Y., Liu W.J., Wang J., Zhang Y., Xu W.P., Tao L.M. (2018). The different effects of natural pyrethrins and beta-cypermethrin on human hepatocyte QSG7701 cells by ROS-mediated oxidative damage. Environ. Sci. Pollut. R.

[23] Syed AAhmed Reza M.I., Kalleti N., Husain A., Singh P., Rath S.K., Gayen J.R. (2023). Evaluation of mutagenic, cytotoxic, mitochondrial dysfunction, apoptotic activity, and acute toxicity of ethanolic extract of Cissus quadrangularis. Toxicology.

[24] Han Y., Zhang H.D., Mu S.C., Wei W., Jin C.Y., Tong C.Y., Song Z.J., Zha Y., Xue Y., Gu G. (2020). Lactate dehydrogenase, an independent risk factor of severe COVID-19 patients: A retrospective and observational study. Aging.

[25] Wilson D.F., Matschinsky F.M. (2023). Integration of eukaryotic energy metabolism: The intramitochondrial and cytosolic energy states ([ATP]f/[ADP]f[Pi]). Int. J. Mol. Sci..

[26] Chen J., Liu W.M. (2023). Lin28a induced mitochondrial dysfunction in human granulosa cells via suppressing LARS2 expression. Cell. Signal..

[27] Qi Z.X., Tong Y.S., Luo H., Chen M., Zhou N., Chen L. (2023). Neuroprotective effect of a Keap1-Nrf2 Protein-Protein Inter-action inhibitor on cerebral Ischemia/Reperfusion injury. Bioorg. Chem..

[28] Li Y.A., Qiu X.L., Lu Z.J., Zhan F.B., Yang M.X., Babu V.S., Li J., Qin Z., Lin L. (2021). Molecular and functional characterization of MST2 in grass carp during bacterial infection. Fish Shellfish. Immun..

[29] Afifi M., Alkaladi A., Zinada O.A.A., Couderchet M. (2017). Alteration in antioxidant genes expression in some fish caught from Jeddah and Yanbu coast as a bio-indicator of oil hydrocarbons pollution. Saudi J. Biol. Sci..

[30] Yang X., Fang Y., Hou J.B., Wang X.J., Li J.Y., Li S.Y., Zheng X.Y., Liu Y., Zhang Z. (2022). The heart as a target for deltamethrin toxicity: Inhibition of Nrf2/HO-1 pathway induces oxidative stress and results in inflammation and apoptosis. Chemosphere.

[31] Diogo B.S., Antunes S.C., Rodrigues S. (2023). Are biopesticides safe for the environment? Effects of pyrethrum extract on the non-target species Daphnia magna. Environ. Toxicol. Pharmacol..

[32] Guo M.H., Li Y.J., Niu S.Y., Zhang R., Shen X., Ma Y., Wu L.Q., Wu T., Zhang T., Tang M. (2024). Oxidative stress-activated Nrf2 remitted polystyrene nanoplastic-induced mitochondrial damage and inflammatory response in HepG2 cells. Environ. Toxicol. Pharmacol..

[33] Li J.J., Jiang H.Q., Zhu Y., Ma Z.J., Li B., Dong J., Xiao C.C., Hu A. (2024). Fine particulate matter (PM2.5) induces the stem cell-like properties of hepatocellular carcinoma by activating ROS/Nrf2/Keap1-mediated autophagy. Ecotoxicol. Environ. Saf..

